# Definition dependent properties of the cortical silent period in upper-extremity muscles, a methodological study

**DOI:** 10.1186/1743-0003-11-1

**Published:** 2014-01-07

**Authors:** Annette AA van Kuijk, Chantal D Bakker, Jan CM Hendriks, Alexander CH Geurts, Dick F Stegeman, Jaco W Pasman

**Affiliations:** 1 Department of Rehabilitation, Radboud University Medical Centre, PO Box 9101, NL-6500 HB Nijmegen, The Netherlands; 2 Health Evidence, Radboud University Medical Centre, Nijmegen, The Netherlands; 3 Neurology, Donders Institute for Brain, Cognition, & Behaviour, Radboud University Medical Centre, Nijmegen, The Netherlands; 4 Faculty of Human Movement Sciences, Research Institute MOVE VU University, Amsterdam, The Netherlands; 5 Libra Rehabilitation & Audiology, Blixembosch, Eindhoven, The Netherlands

**Keywords:** Transcranial magnetic stimulation, Stimulus–response curves, Silent period definition, Stroke

## Abstract

**Background:**

To explore if stimulus–response (S-R) characteristics of the silent period (SP) after transcranial magnetic stimulation (TMS) are affected by changing the SP definition and by changing data presentation in healthy individuals. This information would be clinically relevant to predict motor recovery in patients with stroke using stimulus–response curves.

**Methods:**

Different landmarks to define the SP onset and offset were used to construct S-R curves from the biceps brachii (BB) and abductor digiti minimi (ADM) muscles in 15 healthy participants using rectified versus non-rectified surface electromyography (EMG). A non-linear mixed model fit to a sigmoid Boltzmann function described the S-R characteristics. Differences between S-R characteristics were compared using paired sample *t*-tests. The Bonferroni correction was used to adjust for multiple testing.

**Results:**

For the BB, no differences in S-R characteristics were observed between different SP onset and offset markers, while there was no influence of data presentation either. For the ADM, no differences were observed between different SP onset markers, whereas both the SP offset marker “the first return of any EMG-activity” and presenting non-rectified data showed lower active motor thresholds and less steep slopes.

**Conclusions:**

The use of different landmarks to define the SP offset as well as data presentation affect SP S-R characteristics of the ADM in healthy individuals.

## Background

Transcranial magnetic stimulation (TMS) of the human motor cortex is a noninvasive technique to assess the integrity of the corticospinal motor pathways. Transcranial magnetic stimulation of the primary motor cortex elicits a motor evoked potential (MEP) as an excitatory effect that can be recorded by surface electromyography (EMG) of the target muscles. In pre-activated muscles, TMS also induces a transient suppression of the EMG-activity after the short-latency MEP, i.e. the silent period (SP), as an inhibitory effect [1-4]. Both MEP and SP have been used to predict post-stroke motor recovery [5,6]. Although MEPs are highly predictive with regard to recovery of hand motor function after stroke, their negative predictive value is substantially lower [7]. Both shortened and prolonged SP durations have been proposed as negative prognostic factors for motor recovery after stroke [8]. As such, the SP might be used to optimize the negative predictive value of TMS with regard to post-stroke motor recovery. However, a review on the role of the SP in predicting motor recovery after severe stroke showed rather inconsistent results [9].

Comparison of the studies focusing on the role of the SP in post-stroke motor recovery is difficult due to variability in patients’ characteristics and the time post stroke, but also due to differences in the applied methodology. Indeed, the technique to elicit and assess the SP is all but standardized [9]. Although the SP duration is relatively unaffected by the size of the preceding MEP [10] or the *level* of muscle pre-activation [11-15], it varies with the stimulus intensity used. As a consequence, motor threshold alterations can easily influence the SP duration and may lead to factitious SP changes. This methodological drawback may be significant particularly in clinical practice as threshold changes are commonly encountered in disease states such as stroke [16].

In this perspective, Kimiskidis and associates [17] designed a study to investigate the SP independently from the corticomotor threshold. They constructed stimulus–response curves and fitted the SP data into a sigmoid Boltzmann function. The parameters derived from the Boltzmann function were used for quantitative analysis of the SP. In this way the SP characteristics could be dissociated from the corticomotor threshold. Moreover, the entire stimulus–response curve, quantified by the parameters SP threshold, SP slope, and Max value, could provide more informative and comprehensive estimates of the brain inhibitory mechanisms compared to one single SP duration derived from a certain stimulus intensity. The SP threshold of the stimulus–response curve reflects the stimulus intensity required to activate the most excitable elements of the inhibitory neuronal circuits. The SP slope, provides a general estimate of the increase in SP duration for a given increase in stimulus intensity, indicating the excitability of the pathway. The Max value of the SP curve is an indicator of the peak of the stimulus–response relationship. It reflects threshold for activating all inhibitory interneurons involved in a particular network.

Yet, even if the SP is studied independently of the corticomotor threshold, lack of uniformity regarding the landmarks to define the SP onset and offset may influence its duration [18]. In addition, when the SP is manually (visually guided) assessed, limited inter-rater reliability has been reported [19,20], which is influenced by the way in which the EMG data are presented (e.g. non-rectified versus rectified) [20]. To accurately and reliably determine the SP duration in EMG tracings, we should know what influence the use of different landmarks has on the (variability of the) SP duration. Hence, the primary aim of this study was to study if the use of different landmarks to define the SP onset and offset affects its stimulus (TMS) - response (EMG) characteristics. The ultimate goal was to provide a basis for standardizing the procedure for visually guided manual assessment of SP data in clinical (stroke) practice. Because this study was conducted in the context of assessing patients with upper-extremity paralysis due to stroke, a circular coil was used [21]. Yet, to avoid disease related changes in corticomotor threshold, we conducted this TMS study in healthy subjects. Because proximal and distal upper-extremity muscles may differ in their stimulus–response characteristics [22], stimulus–response curves were constructed for both proximal arm and distal hand muscles.

## Materials and methods

### Subjects

Eighteen healthy individuals participated in this study. Because it has been reported that the threshold for eliciting a SP in upper-extremity muscles on the dominant side is lower than on the non-dominant side [23], only right-handed subjects were included. Handedness was tested with the Edinburgh Handedness Inventory [24]. Subjects with a history of epilepsy, cardiac disorders, pacemaker implantation, craniotomy, psychiatric or neurological diseases were excluded. Pregnant women or individuals using medication with possible effects on the nervous system were excluded as well. Approval of the local Ethics Committee (CMO region Arnhem-Nijmegen reference number 2006/007) was obtained and all participants gave written informed consent.

### EMG-recordings

**Figure 1:**
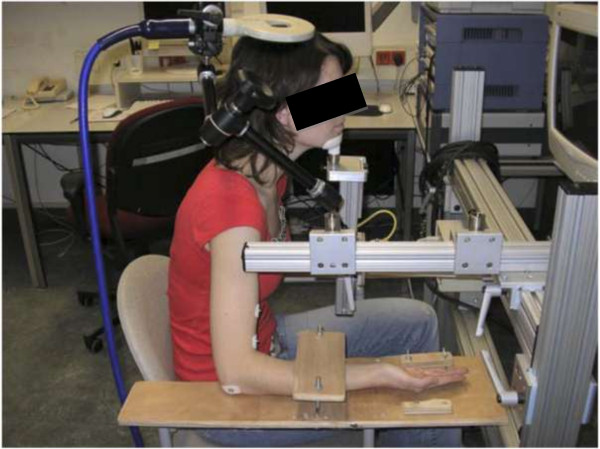
**Position of the participant in a chair with the right arm placed in a fixed frame and a circular coil placed above the vertex.** Written informed consent for publication of the image was obtained from the patient.

Participants were comfortably seated in a chair with their right forearm and hand supinated and supported by a custom built device (Figure 1). The elbow was positioned in 90 degrees flexion. The device restricted any movement of the upper arm, forearm, wrist, and fingers. Bipolar EMG-recordings were obtained using 2 pairs of self-adhesive surface electrodes (Ag-AgCl, solid gel, foam electrodes (35 × 22 millimeters)) placed in a standard tendon belly montage. EMG-signals were recorded using a CED (Cambridge Electronic Design Ltd) data acquisition and amplifier system with a bandpass filter of 20 to 3000 Hz at a display sensitivity of 0.5 microvolt/division (amplifier range 100 millivolt), using a recording time from 150 milliseconds before until 850 milliseconds after each stimulus. The sampling rate was 20.000 samples/second. The EMG data were collected using Spike2 laboratory software (Cambridge Electronic Design Ltd).

First, EMG-activity was recorded from the biceps brachii muscle (BB). The forearm and hand were supinated and EMG-activity of the BB was recorded while participants performed elbow flexion against a fixed frame. Secondly, to obtain isometric abductor digiti minimi (ADM) muscle contractions, right digit V abduction was performed against a fixed frame, while digits II-IV were immobilized by Velcro straps. For measurements of both proximal and distal muscles, participants were instructed to exert maximum force for 3 seconds during 3 trials. The maximal voluntary EMG-activity was defined as the mean EMG-amplitude achieved during these 3 trials.

### Transcranial magnetic stimulation

Transcranial magnetic stimulation of the motor cortex was performed through a 90 millimeter circular coil powered by a Magstim 200 magnetic stimulator (Magstim Company Limited). The vertex was located and marked directly on the scalp. The coil was positioned in a tangential plane near the vertex at approximately 45 degrees to the sagittal line (Mid-central - Cz according to the international 10–20 system of electrode placement) and fixed in this position through a mechanical arm. A counterclockwise inducing current flow was used to activate the left hemisphere.

During the TMS sessions, the participants performed constant isometric muscle contractions at 50% of their maximal voluntary EMG-activity to ensure optimal facilitation of motoneurons [15,25]. Visual feedback of rectified EMG-activity was provided through a 17 inch computer screen placed 1 meter in front of the participants. The EMG target level was presented as a vertical line. The participants were instructed to build up and maintain their muscle activity as close as possible to this level, until they were allowed to relax for 2 seconds after each stimulus. Transcranial magnetic stimulation was delivered 2 seconds after the target activation level was reached. Stimuli were applied with increasing intensities ranging from 20% to 100% (in steps of 5% increments) of the maximum stimulator output. The different stimulus intensities were applied in random order. At each stimulus intensity, 5 consecutive trials were performed with an inter-stimulus interval of approximately 5 seconds. To prevent the occurrence of fatigue the consecutive sessions of different stimulus intensities were separated by at least 30 seconds rest.

### Data analysis

The EMG was recorded in two files. In one file the data were stored after rectification, whereas the raw (non-rectified) data were stored in a different file. At each stimulus intensity, the SP duration was determined from the average of 5 trials. The active motor threshold was defined as the minimum stimulus intensity needed to elicit a recordable SP from the target muscle in at least 3 out of 5 trials. The SP duration was defined as the latency between SP onset and SP offset in the EMG recordings. If no SP could be detected in the averaged data, a duration of 0 milliseconds was assigned.

In both the non-rectified and rectified EMG data, the landmarks to define the SP onset can potentially be defined as stimulus onset, MEP onset, or MEP offset. However, in many healthy individuals, and even more pronounced in patients with stroke, the MEP does not show a clear-cut ending. As a result, the MEP offset is difficult to visually assess. Because the stimulus and MEP onsets appear much more distinctly in the EMG tracings, they were both used to mark the SP onset in this study.

**Figure 2:**
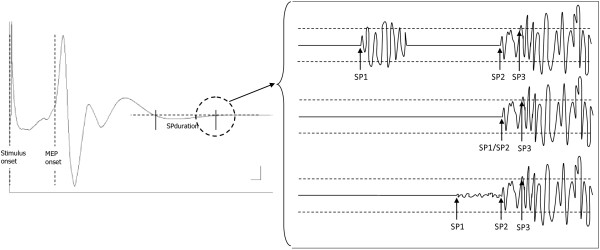
**Landmarks to define the silent period (SP) offset.** SP1: the earliest reoccurrence of any EMG-activity, including burst activity; SP2: the return of continuous EMG-activity, and SP3 the return of continuous EMG-activity to pre-stimulus levels.

As for the SP offset, the landmarks were defined as (1) the earliest reoccurrence of any EMG-activity, including burst activity (i.e. short peaks of reappearing EMG-activity followed by EMG silence) (SP1), (2) the return of continuous EMG-activity (SP2), and (3) the return of continuous EMG-activity to pre-stimulus levels (SP3). In the non-rectified data the pre-stimulus level was determined as the root mean square (RMS) amplitude of the EMG activity during the time period from 150 to 25 milliseconds before stimulus delivery. In the rectified data the pre-stimulus level was determined as the mean amplitude during the same time segment (Figure 2).

### Statistical Methods

The mean SP duration was plotted against stimulation intensity for each participant. Visual inspection of the stimulus–response curves revealed that the relation between these variables had a sigmoid shape. Hence, the data were fitted into a three-parameter sigmoid statistical model (Boltzmann equation):

$$y_{\mathrm{ij}}=\frac{a_{i}}{1+e^{\left(b_{1i}-S_{j}\right)\;/b_{2i}}}+\varepsilon_{\mathrm{ij}}$$

where the three parameters of the function are:and where

1) a_*i*_ reflecting the plateau value of participant *i*

2) b_1*i*_ reflecting the active motor threshold of participant *i*, and

3) b_2*i*_ reflecting the slope parameter (steepness) of participant *i*,

*y*_*ij*_ is the SP duration of participant *i* at stimulus intensity *j*,

*S*_*j*_ is the stimulus intensity *j*, and ε_*ij*_ is the normally distributed residual with mean zero and variance *σ*^2^ of participant *i* at stimulation intensity *j*.

A non-linear mixed regression model was used to fit the individual data to the sigmoid Boltzmann function. The dependent variables were the SP durations of the ADM and BB muscles (*y*) and the independent variable was the stimulus intensity (*S*). The plateau value (*a*) was treated as random effect to allow subject-specific regression coefficients. The slope parameter (*b*_*2*_) and the active motor threshold (*b*_*1*_) were treated as fixed (subject independent) regression coefficients. Differences between the models with random effects and models with fixed effects were tested for statistical significance, using the likelihood ratio test. Note that this test is based on the full likelihood function of the hierarchical models [26]. All parameters were estimated simultaneously together with their standard errors (SEs) as measures of variability, using maximum likelihood methods of the model. Paired-sample *t*-tests were used to test differences in stimulus–response characteristics between the pair wise comparisons of the selected landmarks to define SP onset and offset, as well as between rectified and non-rectified data. The Bonferroni correction was used to adjust for multiple testing. The required 2-tailed significance level was set at 0.05. All statistical analyses were performed with the statistical software package SAS version 8.2 (SAS Institute Inc., North Carolina).

## Results

Seven men and eleven women between 23 and 49 years of age were initially included, however, 3 subjects failed to complete the experiment due to discomfort during the stimulation. Hence, the data of the remaining 15 right-handed participants (6 men, 9 women, mean 32 years, standard deviation 9 years) were used to construct the stimulus–response curves (Table 1).

**Table 1:** Characteristics of the 15 healthy volunteers

		**Number**
Gender	Female	9
	Male	6
Handedness	Right handed	15
(Edinburgh Handedness Inventory)	Left handed	0
Mean age, years (range; SD)	32 (23–49; 9)	

### BB muscle

**Figure 3:**
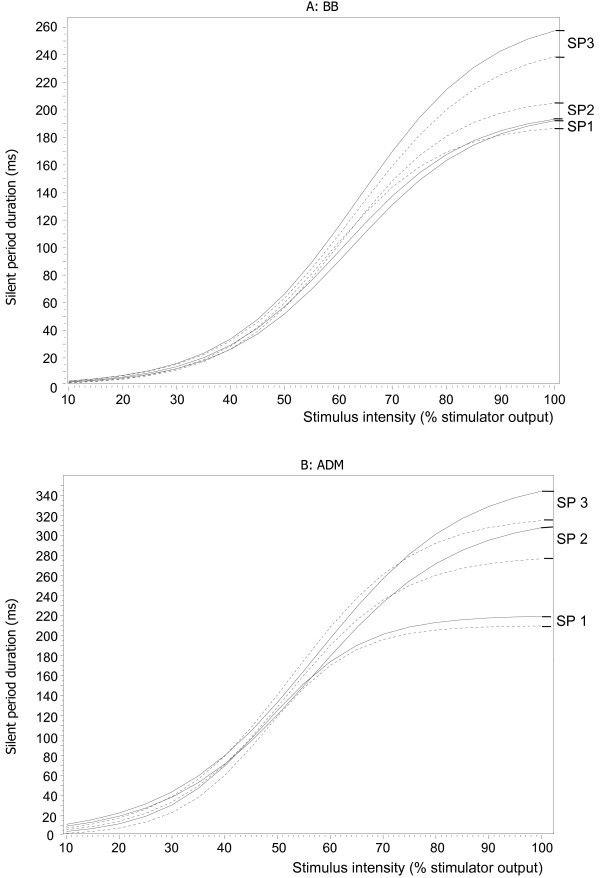
**Average stimulus–response curves (Boltzmann function) of the silent period (SP) obtained from rectified (straight line) and non-rectified (dashed line) EMG of the Biceps Brachii (BB) muscles (A, top) and the Abductor Digiti Minimi (ADM) muscles (B, bottom) in 15 healthy participants.** SP1: the earliest reoccurrence of any EMG-activity, including burst activity; SP2: the return of continuous EMG-activity, and SP3 the return of continuous EMG-activity to pre-stimulus levels.

Figure 3A shows the group averaged stimulus–response curves for the 3 different SP offsets in both rectified and non-rectified EMG of the BB muscle. Table 2 shows the estimated parameters with their SEs for the BB muscle.

**Table 2:** The estimated parameters of the Boltzmann function for the stimulus–response curves of the silent periods in the Biceps Brachii (BB) muscle, using a nonlinear mixed model

	**Rectified EMG**			**Non-rectified EMG**		
**MEP onset**	**SP1 mean (SE)**	**SP2 mean (SE)**	**SP3 mean (SE)**	**SP1 mean (SE)**	**SP2 mean (SE)**	**SP3 mean (SE)**
Plateau value (a) (milliseconds)	0.19 (0.06)	0.20 (0.05)	0.26 (0.06)	0.17 (0.05)	0.20 (0.05)	0.25 (0.05)
Active motor threshold (b1)						
(% stimulator output)	62.90 (2.28)	64.56 (1.41)	65.24 (1.19)	59.46 (1.73)	62.49 (1.03)	65.24 (1.60)
Slope parameter (b2) (%)	12.20 (1.38)	12.23 (0.85)	12.38 (0.70)	10.92 (1.15)	11.27 (0.66)	12.91 (0.92)
SD_res_	0.02	0.02	0.03	0.01	0.02	0.02
**Stimulus onset**						
Plateau value (a) (milliseconds)	0.20 (0.06)	0.20 (0.05)	0.27 (0.06)	0.19 (0.05)	0.21 (0.05)	0.25 (0.05)
Active motor threshold (b1)						
(% stimulator output)	60.69 (2.11)	62.34 (1.35)	63.54 (1.17)	58.06 (1.54)	60.60 (0.95)	63.09 (1.33)
Slope parameter (b2) (%)	11.73 (1.33)	11.80 (0.85)	12.10 (0.72)	10.42 (1.05)	10.61 (0.65)	12.13 (0.82)
SD_res_	0.02	0.02	0.03	0.01	0.02	0.03

*SE*: standard error, *SD*_*res*_: within subject (residual) standard deviation, *SP*: silent period; *SP1*: the earliest reoccurrence of any EMG-activity, including burst activity; *SP2*: the return of continuous EMG-activity, and SP3 the return of continuous EMG-activity to pre-stimulus levels.

With regard to SP onset, using either the MEP onset or the stimulus onset did not show any significant effect on the Boltzmann parameters (Table 2). With regard to SP offset, no significant differences were observed either. Although lower active motor thresholds, less steep slopes, and lower plateau values were observed using both SP1 and SP2 compared to SP3, these differences did not reach statistical significance. Moreover, when SP1 was used to delineate the SP offset, both the active motor threshold and the slope showed relatively large SEs compared to both SP2 and SP3. Again, these differences did not reach statistical significance. In the EMG-tracings of the BB, the landmark to define SP1 was often absent, because a complete EMG-silence could not be seen in many participants.

No significant differences in the estimated stimulus–response parameters were found between the rectified and the non-rectified EMG data.

### ADM muscle

Figure 3B shows the group averaged stimulus–response curves for the 3 different SP offsets in both rectified and non-rectified EMG of the ADM muscle. Table 3 shows the estimated parameters with their SEs for the ADM muscle.

**Table 3:** The estimated parameters of the Boltzmann function for the stimulus–response curves of the silent periods in the Abductor Digiti Minimi (ADM) muscle, using a nonlinear mixed model

	**Rectified EMG**			**Non-rectified EMG**		
**MEP onset**	**SP1 mean (SE)**	**SP2 mean (SE)**	**SP3 mean (SE)**	**SP1 mean (SE)**	**SP2 mean (SE)**	**SP3 mean (SE)**
Plateau value (a) (msec)	0.20 (0.04)	0.30 (0.08)	0.35 (0.08)	0.20 (0.04)	0.26 (0.07)	0.29 (0.08)
Active motor threshold (b1)						
(% stimulator output)	49.43 (0.85)	59.27 (1.17)	59.65 (1.17)	49.48 (0.71)	53.93 (0.94)	54.90 (1.07)
Slope parameter (b2) (%)	9.92 (0.68)	13.62 (0.74)	13.97 (0.74)	8.81 (0.58)	11.40 (0.68)	11.99 (0.75)
SD_res_	0.04	0.04	0.05	0.04	0.03	0.03
**Stimulus onset**						
Plateau value (a) (msec)	0.22 (0.04)	0.32 (0.07)	0.36 (0.08)	0.21 (0.04)	0.28 (0.06)	0.32 (0.07)
Active motor threshold (b1)						
(% stimulator output)	47.33 (0.83)	56.72 (1.14)	57.35 (1.17)	47.69 (0.70)	51.78 (0.91)	52.78 (1.03)
Slope parameter (b2) (%)	9.55 (0.68)	13.45 (0.76)	13.85 (0.77)	8.46 (0.57)	10.95 (0.69)	11.56 (0.75)
SD_res_	0.05	0.04	0.05	0.04	0.03	0.04

*SE*: standard error, *SD*_*res*_: within subject (residual) standard deviation, *SP*: silent period; *SP1*: the earliest reoccurrence of any EMG-activity, including burst activity; *SP2*: the return of continuous EMG-activity, and SP3 the return of continuous EMG-activity to pre-stimulus levels.

Using either the MEP onset or the stimulus onset as the SP onset marker did not show any effect on the Boltzmann parameters (Table 3). As for the SP offset, no significant differences in the plateau values were observed between the selected definitions. However, significant differences were observed in active motor threshold and slope between SP1 on the one hand and both SP2 (active motor threshold, paired-sample *t*-test: p < 0.001; slope, paired-sample *t-*test: p < 0.001) and SP3 (active motor threshold, paired-sample *t-*test: p < 0.001; slope, paired-sample *t-*test: p = 0.002) on the other hand, in the rectified EMG. Similar results were found when the non-rectified EMG data were used. Lower active motor thresholds and less steep slopes could be observed in SP1 compared to SP2 and SP3. No differences in these Boltzmann parameters were observed between SP2 and SP3.

In addition, the non-rectified data showed lower active motor thresholds and less steep slopes compared to the rectified data when either SP2 (active motor threshold, paired-sample *t-*test: p = 0.002; slope, paired-sample *t-*test: p = 0.022) or SP3 (active motor threshold, paired-sample *t-*test: p = 0.015; slope, paired-sample *t-*test: p = 0.143) were used to define the SP offset.

## Discussion

This study demonstrates that the use of different landmarks to define the SP offset as well as the way of data presentation (rectified versus non-rectified EMG) affect the SP stimulus (TMS) - response (EMG) characteristics. In healthy individuals, these effects differ between proximal arm and distal hand muscles. Given the importance of using stimulus–response curves to measure parameters of corticomotor excitability for both prognostication and treatment evaluation after stroke, the ultimate goal of this study was to provide a feasible basis for standardizing the procedure for visually guided manual assessment of SP data in a clinical setting. Among other difficulties affecting the reproducibility of TMS studies [3], variability due to different definitions of SP onset and offset and different data presentation can be overcome by establishing a standard among researches.

### SP onset

In healthy subjects, no differences were found in the stimulus–response characteristics using either the stimulus onset or the MEP onset to mark the SP onset. So both the stimulus onset and MEP onset can be used as a clear-cut SP onset marker in healthy individuals. If either the stimulus onset or the MEP onset is used to mark the SP onset, the duration of the MEP is included in the SP duration. In conditions that lead to a prolonged MEP, this could mask concurrent shortening of the SP duration. In patients with stroke, increased MEP latencies have been found in the sub-acute phase post stroke, shifting towards more normal values with progressive stages of recovery [27,28]. If the stimulus onset is used to define the SP onset, changes in MEP latency might affect the SP duration in stroke patients. To avoid this problem, we propose to use the MEP onset as the best landmark to define the SP onset.

### SP offset

In the BB, it was difficult to accurately define the SP1 offset especially at the lower stimulus intensities. Because a complete EMG-silence was not observed in many participants, the landmark to define SP1 was often absent or at least difficult to define at low stimulus intensities. As a result, the active motor thresholds varied substantially, as can be observed in Table 2. In both the BB and ADM muscles, independent of SP2 or SP3 being used as the offset marker, the active motor threshold increased compared to the method using SP1 to define the offset, whereas the plateau level of the function did not differ between these methods. As a result, the slope parameter of the function increased if the SP duration was measured using SP2 or SP3.

Particularly in the BB, re-occurrence of EMG-activity below pre-stimulus levels was seen before the EMG-activity returned to the pre-stimulus levels, specifically at higher stimulus intensities (80-100%). As a result, if SP2 is used to define the SP offset, lower active motor thresholds and less steep slopes were seen compared to SP3. These differences, however, did not reach statistical significance. This early EMG activity observed in the BB at higher stimulus intensities might be due to fluctuations in contraction force in the period immediately after TMS, such that some responses might have been evoked when the active contraction force was more than the targeted pre-activation level [29]. Because inhibitory inputs to corticofugal neurons are weaker in the proximal muscles compared to the distal hand muscles [15], these EMG bursts may also be due to non-primary motor cortex sources of motoneuron excitation, interrupting the SP in proximal arm muscles.

The SP evoked in the muscles of the upper limb originates largely from activation of cortical inhibitory interneurons, although also spinal mechanisms are involved in the early part [1,12]. While the SP originates primarily in the motor cortex, non-primary motor areas projecting to the motor cortex can influence its duration as well [30]. These cortical and subcortical projections can modulate both the inhibitory and the excitatory interneurons within the primary motor cortex and subsequently change in the balance between excitatory and inhibitory inputs to the intracortical motoneurons of the primary motor cortex. Moreover, next to the non-primary motor areas, the low-level EMG present during the SP might be due to spinal factors including reflex activity evoked by muscle spindle facilitation of elbow flexor motoneurons [31]. As such, these bursts should not be regarded as part of the cortical mechanisms eliciting the SP.

For SP3 the EMG data were analyzed relating the resumption of EMG-activity to the level of pre-stimulus EMG-activity [29]. There are a number of reasons why this might be more informative. First, there is likely to be a considerable degree of variation in single actual force level that is produced by a muscle when individuals attempt to maintain voluntary contraction at a particular magnitude [32]. Second, this method can be easily automated in a computerized algorithm to provide a clear reference of baseline EMG-levels during the assessment of TMS data. From the raters‘ perspective, this simplifies the measurement of the SP duration. We, therefore, consider the resumption of continuous EMG-activity to pre-stimulus levels as the best landmark to define the SP offset for both the BB and ADM in healthy subjects.

### Rectified versus non-rectified EMG

In the ADM, the non-rectified data showed lower active motor thresholds as compared to the rectified data for both SP2 and SP3. Rectifying the data alters the appearance of the data and might reduce the differences between successive data points. As a result, the first resumption of EMG activity below pre-stimulus levels might be more difficult to identify in the rectified data, especially in low-voltage ADM muscle activity. In the BB the pre-stimulus levels of EMG activity are more pronounced compared to the ADM and, therefore, less difficult to identify. However, the raters reported that marker placement was easier to perform in rectified data compared to non-rectified data, especially when using any resumption of continuous EMG activity as the SP offset marker.

### Feasibility from patients’ perspective

Three subjects failed to complete the experiment due to discomfort and pain. In these subjects only the assessment of the ADM muscle was performed. The stimulus–response characteristics obtained from the ADM muscle in these 3 participants did not differ from the results of the other participants. In our study isometric contraction levels of 50% of maximal voluntary EMG-activity have been used to ensure maximal facilitation of the MEP [15,25]. Especially in the BB muscle, the strong muscle twitches associated with high stimulus intensities occasionally interfered with the subjects’ effort to keep the force constant. This has been reported in previous TMS studies as well [17]. These high levels of muscle pre-activation might also be responsible for the discomfort and pain some participants experienced at the higher levels of stimulation intensity. Moreover, several participants experienced fatigue during the TMS assessment and had difficulty maintaining the target EMG-level. In patients with stroke it will be even more difficult to maintain this high level of muscle contraction due to paresis. Although it is well known that voluntary pre-activation of the target muscles is necessary to elicit a SP, there is still controversy on the most effective level of pre-activation [33-36]. Hence, more research is needed to define this optimal pre-activation level in patients with stroke.

## Conclusion

This study indicates that the most feasible method to manually assess the SP duration in healthy individuals is the time interval from either stimulus onset or MEP onset to the return to the level of continuous pre-stimulus EMG-activity in rectified data. Among other difficulties affecting the reproducibility of TMS studies, variability due to different definitions of SP onset and offset can be overcome by establishing a standard among researches. Because the total amount of investigated subjects was limited, and because the method of measuring SP was based on ease of practice rather than on reliability data, there is still not enough evidence to make definitive statements about standardization. Moreover, manually assessing TMS data remains subject to inter-rater variability [20,37]. Measuring MEP and SP characteristics is even more complex in stroke patients and, thus, inter-rater reliability may be of greater concern in this population. This notion implies a critical need for methods that add precision to cortico-spinal tract measurements. Hence, more research is needed to define applicability and reliability in larger populations before this method can be used as the standard in the clinical settings.

## Abbreviations

ADM: Abductor digiti minimi muscle; BB: Biceps brachii muscle; EMG: Electromyography; MEP: Motor evoked potential; RMS: Root mean square; SD: Standard deviation; SDres: Within subject (residual) standard deviation; SE: Standard error; SP: Silent period; SP1: The earliest reoccurrence of any EMG-activity, including burst activity; SP2: The return of continuous EMG-activity; SP3: The return of continuous EMG-activity to pre-stimulus levels; S-R: Stimulus–response; TMS: Transcranial magnetic stimulation.

## Competing interests

The authors declare that they have no competing interests. There is no conflict of interests between the authors and others, and there were no sources of support or funding involved in this study.

## Authors’ contributions

AK participated in the design of the study, carried out the study, and drafted the manuscript, CB participated in the draft of the manuscript, JH participated in the design of the study, performed the statistical analysis, and participated to draft the manuscript. AG and DS both participated in the design of the study as well as in the draft of the manuscript; JP participated in the design and coordination of the study, and drafted the manuscript. All authors read and approved the final manuscript.
